# Psychometric properties of the French-language version of the Coercion Experience Scale (CES)

**DOI:** 10.1186/s12991-019-0230-x

**Published:** 2019-05-17

**Authors:** Philippe Golay, Jérôme Favrod, Stéphane Morandi, Charles Bonsack

**Affiliations:** 1 Community Psychiatry Service, Department of Psychiatry, Lausanne University Hospital and University of Lausanne, Lausanne, Switzerland; 2 General Psychiatry Service, Treatment and Early Intervention in Psychosis Program (TIPP–Lausanne), Lausanne University Hospital and University of Lausanne, Lausanne, Switzerland; 3 Institute of Psychology, Faculty of Social and Political Science, University of Lausanne, Lausanne, Switzerland; 4 La Source, School of Nursing Sciences, HES-SO University of Applied Sciences and Arts of Western Switzerland, Lausanne, Switzerland

**Keywords:** Perceived coercion, Compulsion, Reliability, Validity

## Abstract

**Background:**

The Coercion Experience Scale (CES) was designed to measure the psychological impact of psychiatric coercive interventions. The French-language CES was adapted using a translation/back-translation procedure. It consists originally of 31 items and 6 subscores.

**Aim:**

The goal of this study was aimed to assess the psychometric properties of the French-language CES.

**Method:**

146 inpatients were evaluated. Internal validity was assessed using confirmatory factor analysis. Reliability was estimated using internal consistency coefficients and a test–retest procedure. Convergent validity was estimated using correlations between the AES scores and the Coercion Ladder (CL), the MacArthur’s Admission Experience Survey (AES) and the World Health Organization Quality of Life (WHOQOL-BREF) scale. Discriminatory power was evaluated by comparing the scores of patients undergoing voluntary or compulsory admission.

**Results:**

Although the six-factor original model of the CES showed adequate fit to the data of the French-language version, two factors were almost indistinguishable. A well-defined five-factor alternative was proposed. The CES scores showed good internal consistency. Test–retest reliability varied from good to weak among the five subscores. Correlations between CES and CL, AES and WHOQOL scores suggested good convergent validity for most scores. Two CES scores were significantly higher among patients subject to compulsory psychiatric hospital admission than among those admitted voluntarily.

**Conclusions:**

Overall, the French-language version of the CES is a usable tool to study different aspects of perceived coercion.

## Introduction

In psychiatric treatments, the rationale behind coercion is to protect people with mental disorders and improve their health [1]. However, evidence of any patient benefits from compulsory inpatient admission is considered scarce by some authors [2]. Studies has suggested that coercion may have severe, long-lasting negative effects on patients, such as worse quality of life [3], lower treatment adherence [4], potentially cause trauma or trigger past-trauma [5] and lower satisfaction with care [6]. Another concerning effect was that coercion seemed to increase the use of future coercive measures [7–9].

Perceived coercion is related to several aspects, the obvious being the formal coercive measures or the patient’s legal status at admission. Among other factors, the amount of information shared with the patient, the participation in medical decision making and the lack of knowledge about legal issues also contributes to perceived coercion [10]. Less formal forms of coercion such as leverage can also contribute to that phenomenon [11]. Not surprisingly, even voluntary patients are subjected to perceived coercion [12, 13]. This is important because it has been shown that the patients’ level of perceived coercion can have damaging effects on the patient’s perception of the therapeutic relationship [14] and can influence negatively their prognoses even more than the coercive measure itself [15].

To the best of our knowledge, there is only one specific French-language tool available for the study of perceived coercion which is the MacArthur Admission Experience Survey short form (AES) [16]. Although easy to use and very short, the AES does not cover all aspects of coercion. The AES only refers to the hospital admission process and is therefore not suitable to measure the impact of other coercive interventions [17].

The Coercion Experience Scale (CES) was based on the observation that there was only very few instruments that could be used in trials in order to evaluate the patients’ subjective experience of coercive interventions in psychiatry during hospitalisations [17]. According to the authors, an adequate tool should also be “*applicable to more than one intervention in order to detect differences between two or more coercive interventions, reflect the ethical considerations referring to the restriction of human rights, cover a wide range of interindividual highly varying stressors, and account for the specific psychiatric context*” [17]. Therefore, restrictions of human rights and stressors were paramount theoretical considerations during the development of the questionnaire.

The rarity of research tools makes investigation of coercion in French-speaking countries difficult. In order to address this problem, the adaptation of the French-language CES was undertaken. The aim of this study was to assess the psychometric properties of this much-needed tool.

## Methods

### Participants

A total of 146 patients were recruited during their hospitalisation in Lausanne University Hospital’s Department of Psychiatry (Table 1). Patients were approached by a research assistant in the presence of their attending doctor or nurse. After a period of consideration, people who agreed to participate signed the consent form and were interviewed individually. Written informed consent was obtained from all participants. Mean age was 41.8 years old and a slight majority of participants were women. The average level of general functioning, as assessed using the Global Assessment of Functioning (GAF) scale, was 41.8 (SD = 12.9) and about one-third of patients were admitted involuntarily, according to their caregivers. Twenty-one percent of the patients had a GAF score higher than 50. The GAF score of involuntary patients was significantly lower (*t*(136) = 3.165, *p* < 0.002, *d* = 0.58). The majority of patients were born in Switzerland and all participants were either native French speakers or proficient with French. Primary diagnoses based on the International Statistical Classification of Diseases and Related Health Problems 10th Revision (ICD-10) were 35.6% schizophrenia, 32.9% depression, 11.6% personality disorder, 5.5% mania, 4.8% anxiety and stress-related disorders, 5.5% drug use and 4.1% alcohol use.

**Table 1:** Participants’ characteristics

	Total *N *= 146
Age, mean (SD)	41.8 (12.8)
Gender, female, % (*n*)	51.4 (75)
Global Assessment of Functioning (GAF), mean (SD)	41.8 (12.9)
Admission mode, involuntary, % (*n*)	30.1 (44)
Born in Switzerland, % (*n*)	68.5 (100)
Primary diagnostic, % (*n*)	
Schizophrenia	35.6 (52)
Depression	32.9 (48)
Personality disorder	11.6 (17)
Mania	5.5 (8)
Anxiety and stress-related disorder	4.8 (7)
Drug use	5.5 (8)
Alcohol use	4.1 (6)

### Measures

#### Coercion Experience Scale (CES)

The CES [17] is a 31-item scale designed to measure patients’ experiences of coercive measures. The scale was developed in German and the items were translated and published into English [17]. The first two items are 0–100 visual analogue scales designed to evaluate the extent to which patients remember coercive measures (item 1) and the extent to which these were considered stressful (item 2). All 29 other items are five-point Likert-type scales. Six dimensions were identified: *Humiliation, Physical adverse effects, Interpersonal separation, Negative environmental influences, Fear* and *Coercion.* The French-language version of the CES was first translated from English to French (JF) and then back-translated in German by an independent professional translator. No noteworthy changes were required and the content of the French translation of the CES items was approved by the original authors.

#### Coercion Ladder

The Coercion Ladder [18] was originally adapted from the Cantril Ladder [19]. It is a visual analogue tool on which the patient is asked to mark the degree of perceived coercion on a scale of 1 (Minimum use of coercion—*I came totally on my own will and initiative*) to 10 (Maximum use of coercion). The Coercion Ladder’s test–retest reliability is satisfactory (*r* = 0.77; ICC [1, 2] = 0.77) [16].

#### MacArthur Admission Experience Survey short form

The Admission Experience Survey (AES) short form developed for the MacArthur Coercion Study was derived from a structured interview (the *MacArthur Admission Experience Interview*) so that patients’ perceptions of psychiatric hospital admission could be obtained rapidly using a paper and pencil. The AES was translated and validated into French [16]. This 16 items questionnaire allows the computation of three subscales and a total score. The *Perceived Coercion* score focuses on freedom, choice, initiative, control and influence over coming into hospital; the *Negative Pressures* score focuses on being forced, threatened or physically forced to come into hospital and the *Voice* score focuses on having a chance to voice an opinion about coming into hospital [16].

#### World Health Organization Quality of Life (WHOQOL-BREF)

The WHOQOL-BREF [20] was derived from data collected with the WHOQOL-100. It includes 26 Likert-type items and four scores related to quality of life can be computed: *physical health, psychological, social relationships* and *environment*.

### Procedure

To assess the internal validity of the French-language CES scores, we used Confirmatory Factor Analysis (CFA). We tested the original six-factor CES scoring model by loading items 3, 5, 7, 9, 11, 14, 17, 20, 22, 24, 25, 29, 30 and 31 onto the *Humiliation factor,* items 13, 21, 23 and 26 onto the *Physical adverse effect factor,* items 4 and 8 onto the *Interpersonal separation factor*, items 12, 15, 16, 18 and 28 onto the *Negative environmental influences factor*, items 19 and 27 on the *Fear factor* and items 6 and 10 on the *Coercion factor*. Two alternative final models were estimated: a five correlated first-order factor model and a higher-order variant with a general coercion factor on top of the five first-order factors.

The reliability of the French-language CES scores was assessed using a test–retest approach with an interval of between 2 and 14 days; 43 patients participated in the retest. Internal consistency estimates were also computed on the basis of the first assessment. To estimate convergent validity, several indicators were used to study the relationship between CES scores and other scales. We hypothesised that the CES Humiliation/coercion score would positively correlate with the Coercion Ladder score, the AES Perceived Coercion and Total scores and with the *CES’s* second item (stress measured on a 0–100 scale). We also expected a negative correlation with the AES Voice score. We hypothesised that the CES Physical adverse effect score would be negatively correlated with the WHOQOL-BREF Physical score. We hypothesised that the CES Interpersonal separation score would be positively correlated to the AES Negative pressure score and negatively correlated to the AES Voice score. We hypothesised that the Negative environmental influence score would be negatively correlated to the WHOQOL-BREF environmental score. We hypothesised that the Fear score would be positively correlated to the CES stressing experience item and the Negative Pressure score and negatively correlated to the WHOQOL-BREF environmental score. Finally, we assessed the divergent validity under the hypothesis that no CES score should be correlated to the recall of the coercive measure (CES’s first item on a 0–100 scale).

### Statistical analysis

#### Internal validity

For CFA, item data were treated as categorical ordinals and the models were estimated using a robust-weighted least squares estimator with adjustments for the mean and variance (WLSMV). The two alternative final models (correlated first-order versus higher order) were compared with a robust Chi-square test using the DIFFTEST procedure. Several indicators of model fit were used: the Root Mean Square Error of Approximation (RMSEA), the Comparison Fit Index (CFI) and the Tucker–Lewis fit Index (TLI). RMSEA values ≤ 0.06 and CFI and TLI values ≥ 0.95, were interpreted as good fits, whereas RMSEA values ≤ 0.08 and CFI and TLI values ≥ 0.90 were considered as indicating acceptable fit [21].

#### Reliability

The reliability of the CES subscales was estimated using McDonald’s model-based Omega (*ω*) [22] and Cronbach’s alpha (*α*) coefficients. The Cronbach alpha coefficient assumes unidimensionality, tau-equivalence (same factor loadings), no residual correlations and is notoriously biased when the number of items is small. Therefore McDonald’s model-based Omega estimates provide a more reliable information about reliability. The test–retest reliabilities were estimated using both Pearson and intraclass correlation coefficients using a two-way random-effects model and the absolute agreement definition (ICC [1, 2]). Reliability coefficients above 0.70 were considered satisfactory; above 0.80 were considered good and above 0.90 were considered excellent [22, 23].

#### Convergent validity

The convergent validity coefficients between the French-language CES scores and the other scales were estimated using Pearson correlation coefficients. Because under Classical Test Theory, the upper bound of validity coefficients is limited to the square root of the score reliabilities; the acceptable range is usually lower than for reliability coefficients. Correlation coefficients between 0.40 and 0.60 were considered as good and any values higher than 0.30 (a medium effect size, according to Cohen [24]) as satisfactory.

#### Discrimination

To test whether the French-language CES could discriminate between voluntarily and involuntarily admitted patients, their average scores were compared using an independent sample Student *t* test. Our hypothesis was that the latter group would report higher levels of coercion. All statistical tests were two-tailed, and a significance level was set at *α* = 0.05. All statistical analyses were performed using the Mplus statistical package (version 7.4) and IBM SPSS 25.

## Results

### Internal validity

The six-factor model fit was satisfactory (*χ*^2^ = 657.175; *df* = 365, *p* < 0.001, RMSEA = 0.074, CFI = 0.956, TLI = 0.951). However, the *Humiliation factor and Coercion* factors were almost perfectly correlated (*r* = 0.958; Table 2) and the loadings of two items (#13 & #15) were not significant. These two factors were merged into a Humiliation/coercion factor. Items that did not load on their respective factors were also excluded.

**Table 2:** Six-factor model of the CES scale

	Standardised loadings					
A—Humiliation factor						
#3. Adverse effects on your human dignity	0.765*					
#5. Restrictions of your ability to move	0.839*					
#7. Restrictions of your freedom to decide things	0.869*					
#9. Restrictions of my ability to move	0.890*					
#11. Restrictions of my freedom to decide things	0.877*					
#14. I felt my dignity taken away	0.714*					
#17. I had to obey the orders of others	0.710*					
#20. Others made decisions on me	0.848*					
#22. I did not know what to expect	0.663*					
#24. I could not understand why I was being treated that way	0.815*					
#25. I could not move freely	0.809*					
*#*29. I felt dealt like an animal	0.839*					
#30. I feared the measure would last forever	0.835*					
#31. My wishes were not taken into account	0.727*					
B—Physical adverse effects factor						
#13. I suffered pain	0.128					
#21. Passing urine or defecating was shameful	0.955*					
#23. Passing urine or defecating was uncomfortable	0.780*					
#26. Having to attend to washing whilst being observed by aid of staff	0.981*					
C—Interpersonal separation factor						
#4. Restrictions of your ability to have contact with staff	0.895*					
#8. Restriction of contact with staff	0.895*					
D—Negative environmental influences factor						
#12. I feared not getting enough air	0.709*					
#15. I was not able to sleep well	0.204					
#16. The decor or lighting of the room were unpleasant	0.600*					
#18. The room was too cold or too warm	0.640*					
#28. Poor condition of air in the room	0.798*					
E—Fear factor						
#19. I was afraid I would be killed	0.854*					
#27. I was afraid I would die	0.854*					
F—Coercion factor						
#6. Experience of coercion	0.908*					
#10. Applied coercion	0.908*					

Factor correlation	A	B	C	D	E	F
A—Humiliation factor	1.000					
B—Physical adverse effects factor	0.547*	1.000				
C—Interpersonal separation factor	0.721*	0.492*	1.000			
D—Negative environmental influences factor	0.590*	0.606*	0.566*	1.000		
E—Fear factor	0.365*	0.623*	0.234	0.565*	1.000	
F—Coercion factor	0.958*	0.449*	0.674*	0.493*	0.260*	1.000

* *p* < 0.05

Both the higher-order five-factor model and the first-order five-factor model showed adequate fit to the data (Higher order : *χ*^2^ = 606.723; *df* = 321, *p* < 0.001, RMSEA = 0.078, CFI = 0.958, TLI = 0.954; first-order five factor: *χ*^2^ = 585.878; *df* = 316, *p* < 0.001, RMSEA = 0.076, CFI = 0.960, TLI = 0.956). Direct comparison between the two models indicated that the correlated five-factor solution was preferable to the higher-order variant (Δ*χ*^2^ = 23.722; Δ*df* = 5; *p* < 0.001). All factor loadings were significant (Table 3). The French final version is presented in Table 4.

**Table 3:** Final five-factor model of the French-language CES scale

	Standardised loadings				
A—Humiliation/coercion factor					
#3. Adverse effects on your human dignity	0.766*				
#5. Restrictions of your ability to move	0.840*				
#6. Experience of coercion	0.849*				
#7. Restrictions of your freedom to decide things	0.868*				
#9. Restrictions of my ability to move	0.887*				
#10. Applied coercion	0.900*				
#11. Restrictions of my freedom to decide things	0.876*				
#14. I felt my dignity taken away.	0.713*				
#17. I had to obey the orders of others	0.710*				
#20. Others made decisions on me	0.848*				
#22. I did not know what to expect	0.663*				
#24. I could not understand why I was being treated that way	0.815*				
#25. I could not move freely	0.807*				
*#*29. I felt dealt like an animal	0.839*				
#30. I feared the measure would last forever	0.834*				
#31. My wishes were not taken into account	0.728*				
B—Physical adverse effects factor					
#21. Passing urine or defecating was shameful	0.953*				
#23. Passing urine or defecating was uncomfortable	0.777*				
#26. Having to attend to washing whilst being observed by aid of staff	0.990*				
C—Interpersonal separation factor					
#4. Restrictions of your ability to have contact with staff	0.895*				
#8. Restriction of contact with staff	0.895*				
D—Negative environmental influences factor					
#12. I feared not getting enough air	0.704*				
#16. The decor or lighting of the room were unpleasant	0.593*				
#18. The room was too cold or too warm	0.633*				
#28. Poor condition of air in the room	0.792*				
E—Fear factor					
#19. I was afraid I would be killed	0.854*				
#27. I was afraid I would die	0.854*				
Excluded items					
#13. I suffered pain	–				
#15. I was not able to sleep well	–				

Factor correlation	A	B	C	D	E
A—Humiliation/coercion factor	1.000				
B—Physical adverse effects factor	0.545*	1.000			
C—Interpersonal separation factor	0.718*	0.478*	1.000		
D—Negative environmental influences factor	0.588*	0.603*	0.563*	1.000	
E—Fear factor	0.353*	0.599*	0.234*	0.571*	1.000

Descriptive statistics of the first two scales and final five subscales	Mean	SD	Min	Max	
Visual analogue scale to evaluate the extent to which patients remember coercive measures (item 1)	72.23	34.69	0	100	
Visual analogue scale to evaluate the extent to which patients considered coercive measures stressful (item 2)	40.72	37.403	0	100	
A—Humiliation/coercion factor	22.34	18.07	0	64	
B—Physical adverse effects factor	1.85	2.85	0	12	
C—Interpersonal separation factor	1.71	2.23	0	8	
D—Negative environmental influences factor	3.36	3.62	0	16	
E—Fear factor	1.17	2.09	0	8	

*SD* standard deviation

* *p* < 0.05

**Table 4:**
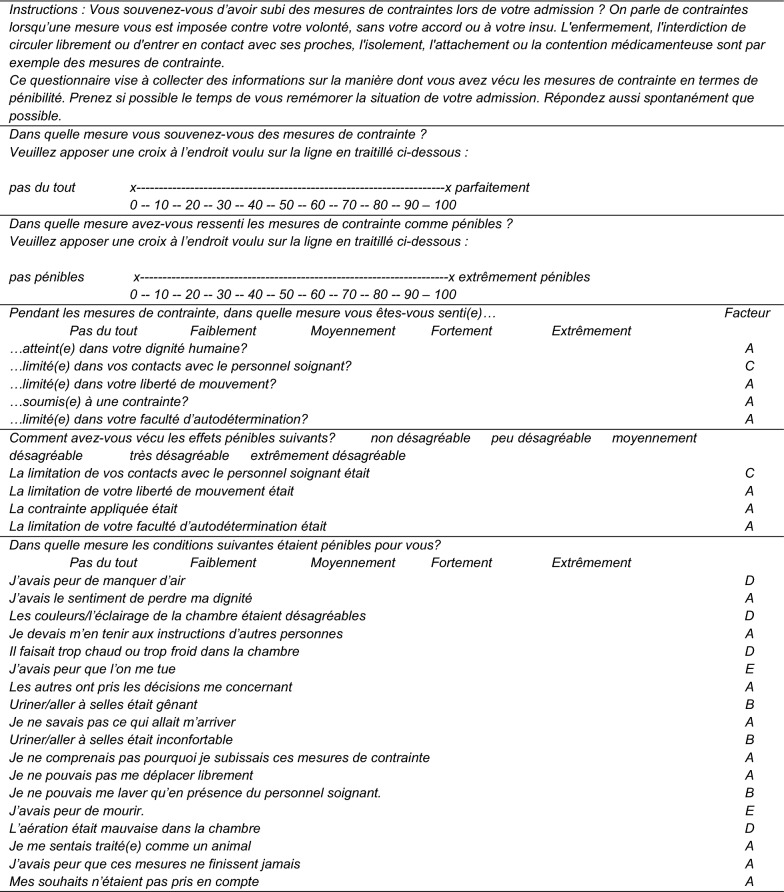
French-language final version of the CES

*A *=* Humiliation* – *coercition, B *=* éléments physiques adverses, C *=* Séparation interpersonnelle, D *=* Influences environnementales négatives, E *=* Peur. Items exclus: J’avais des douleurs; Je dormais mal*

### Reliability

Internal consistency estimates (Table 5) were satisfactory to excellent [22, 23]. However, test–retest reliability estimates were markedly lower with the exception of the Humiliation/coercion subscale score. Comparisons between scores from the first and second assessments revealed no significant changes. There was no significant difference between participants who were included or not in test–retest reliability analysis with regard to general functioning (*t*(136) = − 0.150, *p* = 0.881) and diagnosis (Fisher’s Exact Test, *p* = 0.511).

**Table 5:** Reliability of the French-language version CES scores

	Internal consistency (*N* = 146)		Test–retest reliability (*N* = 43)	
	McDonald’s *ω*	Cronbach’s *α*	Pearson’s *r*	ICC (2,1)
Humiliation/coercion subscale	0.974	0.948	0.896	0.896
Physical adverse effects subscale	0.867	0.681	0.480	0.467
Interpersonal separation subscale	0.908	0.836	0.644	0.643
Negative environmental influences subscale	0.806	0.637	0.658	0.650
Fear subscale	0.860	0.689	0.617	0.586

^*^ = *p* < 0.05

### Convergent and divergent validity

The CES Humiliation/coercion score was positively correlated with the Coercion Ladder score, the AES Perceived Coercion and Total scores and with the *CES’s* second item (Table 6). We also observed a negative correlation with the AES Voice score. The CES Physical adverse effect score was negatively correlated with the WHOQOL-BREF Physical score. The CES Interpersonal separation score was positively correlated to the AES Negative pressure score and negatively correlated to the AES Voice score. The Negative environmental influence score was negatively correlated to the WHOQOL-BREF environmental score. Finally, the Fear score was negatively correlated to the WHOQOL-BREF environmental score but was not significantly related to the CES stressing experience item and the AES Negative Pressure score. Concerning divergent validity, no CES score was significantly correlated to the recall of the coercive measure.

**Table 6:** Convergent validity of the French-language version CES scores

	Coercion Ladder	Coercion—AES				Quality of life—WHOQOL		CES additional items	
		Perceived Coercion score	Negative pressures score	Voice score	Total score	Physical score	Environmental score	Stressing experience (item 2)	Recall of coercive measure (item 1)
Humiliation/coercion subscale	0.718*	0.565*	0.661*	− 0.587*	0.694*	0.019	− 0.293*	0.679*	− 0.041
Physical adverse effects subscale	0.193*	0.091	0.180*	− 0.122	0.186*	− 0.195*	0.197*	0.197*	0.069
Interpersonal separation subscale	0.357*	0.282*	0.392*	− 0.312*	0.407*	− 0.040	− 0.127	0.474*	0.132
Negative environmental influences subscale	0.312	0.239*	0.218*	− 0.169	0.245*	− 0.181*	− 0.207*	0.289*	− 0.044
Fear subscale	0.107	0.016	0.110	− 0.061	0.074	− 0.084	− 0.249*	0.096	− 0.056

^*^ = *p* < 0.05

### Discrimination according to admission status

Involuntarily admitted patients scored higher that patients admitted voluntarily on the *Humiliation/coercion* score (*t*(137) = − 3.674, *p* < 0.001, *d* = 0.68) and the *Interpersonal separation* score (*t*(63.449) = − 2.626, *p* = 0.011, *d* = 0.51). However, no statistically significant differences were revealed for the *Physical adverse effects, Negative environmental influence* and *Fear* scores.

## Discussion

Investigation of the CES’ internal structure revealed that a five-factor model including a *Humiliation/coercion* factor, a *Physical adverse effects* factor, an *Interpersonal separation* factor, a *Negative environmental influences* factor and a *Fear* factor was the most adequate. The *Humiliation* and the *Coercion* factors were almost indistinguishable. The original CES factor structure was assessed on the data of 102 patients using exploratory factor analysis and Varimax orthogonal rotation. Therefore, factors correlations were not estimated but were rather fixed to zero which could explain our findings. The comparison of the higher-order and the correlated five-factor models suggested that the source of factors correlation was not unitary. Hence, the computation of a total coercion score was not warranted. This is in line with the authors of the CES who did not propose a total coercion score [17]. Pain and sleep items did not contribute to the original factors in this study. However, it is important to note that the factor construct of the CES could be affected to some extent by the sample characteristics (e.g. diagnosis, severity of psychiatric symptoms and duration of interventions). Another hypothesis is that pain and sleep could be driven by psychosomatic issues independently of psychiatric coercive interventions.

Comparisons between scores from the first and second assessments revealed no significant changes. Internal consistency estimates were satisfactory for all subscores. However, test–retest reliability was much lower with the exception of the *Humiliation/coercion* subscale score, which demonstrated very high reliability across all estimates. Because internal consistency estimates were shown to be good and the temporal stability of some of the CES scores was only modest, this pattern of findings lead us to believe that the poor test–retest reliability of some of the CES scores may be partially explained by the inherent variability of the constructs in hospital context rather than by poor item and scale construction. The latter would likely have prevented such levels of internal consistency.

Correlations between the French-language AES scores and the Coercion Ladder score, and the Coercion Experience Scale were globally in line with expectations, suggesting that the French-language version of the AES provided a valid measure of different aspects of perceived coercion. As for reliability, the *humiliation/coercion* subscale score was also associated with the higher correlations. Contrary to our hypothesis, the *Fear* score was not significantly related to the CES stressing experience item and the AES Negative Pressure score. It is worth noting that this subscore was based on only two items that were essentially based on fear of death and not fear in general. Examination of the weighted means of the five subscale scores revealed that this dimension was scored lower than any other subscales. At this stage, it remained difficult to ascertain whether the *Fear* subscore had limited validity or if it was not particularly relevant in our actual sample. CES subscores were not related to the quality of recall of the coercive measure. That suggested that patients with better memory of coercive measures did not systematically amplified their scores and vice versa.

Finally, two scores (*humiliation/coercion* and *interpersonal separation*) derived from the French-language CES were able to discriminate between patients who had been voluntarily and involuntarily admitted to hospital, which in part confirmed our hypothesis. However, some negative consequences of hospitalisation such as physical adverse effects, negative environmental influence and fear did not seem related to admission status and may be related to characteristics of inpatient stay in general.

Our study has several limitations that could be the focus of future studies. First, our study did not take diagnostics into account. Further research may include distinct diagnostic groups (e.g. people diagnosed with depression versus schizophrenia). Second, based on their GAF score, a small number of patients could be considered as relatively healthy given they were hospitalised. However, the GAF score of involuntary patients was significantly lower and they experienced more coercion. Third, this study is mainly cross-sectional and a longitudinal design may be used to examine the CES’ sensitivity to change after psychosocial interventions. Whilst involuntary psychiatric treatments aim to protect people with mental disorders [1], the evidence for patients’ benefits of inpatient compulsion could be considered scarce [2] and coercive measures may have severe negative effects [15]. There are several potential benefits of using the CES in research or clinical practice. From a clinical standpoint, the CES has been described as a potential screening instrument “*for patients who need support after coercive interventions to prevent consequences from traumatic experiences*” [17]. The CES can also be used to monitor and compare different clinical settings and interventions. The respect of users’ preferences and needs are part of the foundation of patient-centred care [25]. This approach promotes recovery and tries to foster engagement in treatment [26] and care efficiency [27]. Following this perspective, the CES will allow us to test whether shared decision making (defined as “*an approach where clinicians and patients share the best available evidence when faced with the task of making decisions, and where patients are supported to consider options, to achieve informed preferences*” [28]) can diminish perceived coercion and ameliorate patients’ prognosis and well-being.

## Conclusions

The French-language version of the CES demonstrated adequate psychometric properties. The *humiliation/coercion* score had particularly strong reliability, validity and discrimination between voluntarily and involuntarily admitted patients. The *Fear* subscore may nevertheless warrant more cautious interpretation given the partial fulfilment of our convergent validity’s expectations. We hope the availability of the CES will promote further research projects on this topic in French-speaking countries and lead us to a better understanding of the factors influencing patients’ perceptions of coercion.

## Data Availability

The data sets generated and analysed during the present study are not publicly available because their public archiving was not explicitly authorised by the ethics committee. Nevertheless, anonymous data are available from the corresponding author on reasonable request.

## Abbreviations

CES: Coercion Experience Scale
CL: Coercion Ladder
AES: MacArthur’s Admission Experience Survey
WHOQOL-BREF: World Health Organization Quality of Life
GAF: Global Assessment of Functioning
ICD-10: International Statistical Classification of Diseases and Related Health Problems 10th Revision
CFA: confirmatory factor analysis
RMSEA: Root Mean Square Error of Approximation
CFI: Comparison Fit Index
TLI: Tucker–Lewis fit Index
ICC: intraclass correlation coefficient
